# Engineered Electrochemiluminescence Biosensors for Monitoring Heavy Metal Ions: Current Status and Prospects

**DOI:** 10.3390/bios14010009

**Published:** 2023-12-22

**Authors:** Yuanyuan Chen, Hui Jiang, Xiaohui Liu, Xuemei Wang

**Affiliations:** State Key Laboratory of Digital Medical Engineering, School of Biological Science and Medical Engineering, Southeast University, Nanjing 210096, China; 230239535@seu.edu.cn (Y.C.); sungi@seu.edu.cn (H.J.); 101013182@seu.edu.cn (X.L.)

**Keywords:** electrochemiluminescence, metal ions monitoring, nanomaterials, biosensors

## Abstract

Metal ion contamination has serious impacts on environmental and biological health, so it is crucial to effectively monitor the levels of these metal ions. With the continuous progression of optoelectronic nanotechnology and biometrics, the emerging electrochemiluminescence (ECL) biosensing technology has not only proven its simplicity, but also showcased its utility and remarkable sensitivity in engineered monitoring of residual heavy metal contaminants. This comprehensive review begins by introducing the composition, advantages, and detection principles of ECL biosensors, and delving into the engineered aspects. Furthermore, it explores two signal amplification methods: biometric element-based strategies (e.g., HCR, RCA, EDC, and CRISPR/Cas) and nanomaterial (NM)-based amplification, including quantum dots, metal nanoclusters, carbon-based nanomaterials, and porous nanomaterials. Ultimately, this review envisions future research trends and engineered technological enhancements of ECL biosensors to meet the surging demand for metal ion monitoring.

## 1. Introduction

Over the past few years, the environmental crisis caused by heavy metal ion pollution has reached a critical juncture [1], driven primarily by the rapid development of industries such as mineral extraction, metal smelting, and industrial manufacturing [2]. Continuous releases of heavy metal ions into the environment have resulted in widespread contamination of soils and water bodies globally [3,4]. These pollutants exhibit a ubiquitous distribution and high toxicity, increasingly impacting the integrity of soils and water bodies. Simultaneously, with the rise in the use of fertilizers, feed additives, and cultivation periods, there is a significant upward trend in the concentrations of heavy metal pollution in soils and water bodies, posing severe threats to the livestock industry [5]. Metal ions are not easily degraded by nature and accumulate through the food chain, so they not only pose a long-term threat to the environment, but also play a vital role in organisms (Table 1). Copper (Cu), zinc (Zn), cobalt (Co), and iron (Fe) are considered essential trace elements for human health [6]. However, when they accumulate in the body beyond certain levels, these common heavy metal ions can exhibit pathogenic effects [7]. Specifically, excessive copper intake may lead to Wilson’s disease, characterized by liver abnormalities and neurological issues; prolonged high-dose zinc intake can cause copper deficiency, resulting in anemia, immune system problems, and neurological disorders; excessive cobalt intake may be associated with cardiac problems, affecting the cardiovascular system and other aspects of health; and overconsumption of iron may lead to hemochromatosis, causing damage to the liver, heart, and pancreas, manifested by symptoms such as fatigue and abdominal pain. Copper, as an example, is one of the common elements in the Earth’s crust, found in natural minerals, and extensively used in power transmission, pipeline manufacturing, and automotive components [8,9]. In organisms, copper ions are also coenzymes of some important enzymes, which are involved in a variety of redox reactions. However, when copper ions are ingested or absorbed in excess, it may lead to acute or chronic copper poisoning, and excessive copper ions may also lead to neurological problems, such as anxiety, depression, neuropathy, and cognitive dysfunction [10,11,12]. In addition, certain heavy metals such as mercury (Hg) [13,14,15], cadmium (Cd) [16], lead (Pb) [17,18], and the metalloid arsenic (As) [19,20,21] have significant biotoxicity, and are considered toxic and harmful at low concentrations.

A variety of detection techniques have been developed, such as atomic absorption spectroscopy (AAS) [22], X-ray fluorescence spectroscopy (XRF) [23], inductively coupled plasma mass spectrometry (ICP-MS) [24], and ultraviolet-visible (UV) spectroscopy [25], which have been widely used to detect low-concentration metal ions at the nanogram and picogram levels [26]. While these methods offer outstanding sensitivity in metal ion detection, they come with the drawback of involving complex operational procedures and the need for costly specialized equipment. Furthermore, their bulkiness makes them unsuitable for on-site detection.

Compared with other methods, electrochemiluminescence (ECL) has unique advantages, including high sensitivity, low cost, and easy operation [27,28]. In particular, the emergence of ECL biosensors based on nucleic acids, antigens/antibodies, and microorganisms has provided new hope for the detection of metal ions [29], based on the fact that the ECL biosensor can be biodegradable, highly selective, and the analytical equipment is miniaturized [30]. It has been reported that many ECL biosensors have been identified and used for the detection of metal ions such as Pb^2+^, Cu^2+^, Hg^2+^, and Cr^3+^. In addition, the development and improvement of nanotechnology have provided more attractive advantages for ECL technology, that is, the high specific surface area, conductivity, biocompatibility, and flexibility of nanomaterials. The integration of nanomaterials and biomaterials in ECL biosensors leverages the exceptional attributes of nanomaterials and the binding capabilities of biomaterials. This combination renders ECL biosensors a prime candidate to fulfill the requirements of metal ion analysis [31].

**Scheme 1:**
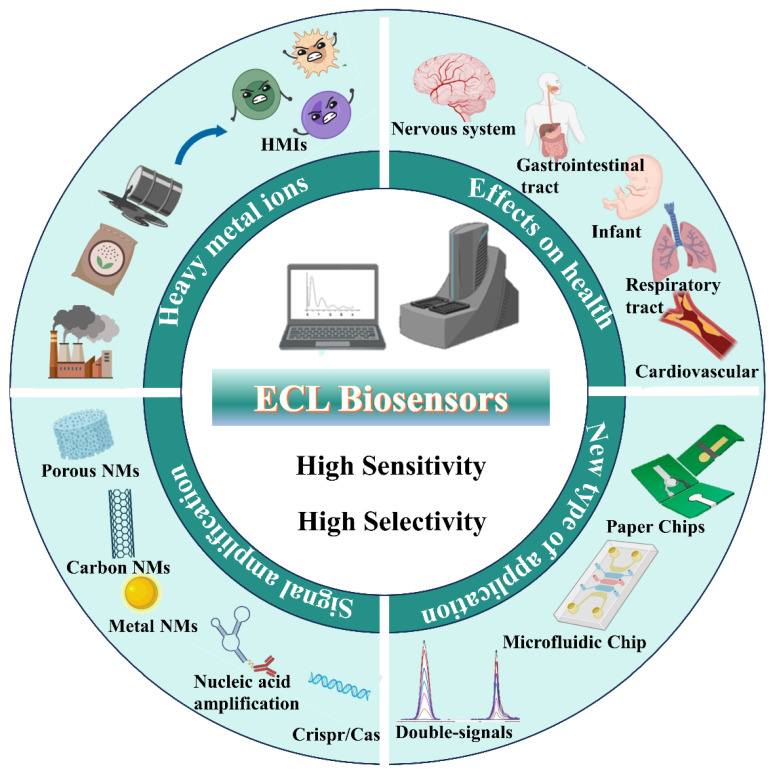
ECL biosensors engineered to monitor heavy metal ions originating from diverse sources.

Given the engineered potential of ECL biosensors in heavy metal ion monitoring (as depicted in Scheme 1), this paper systematically assesses the advancements in ECL biosensors for monitoring heavy metal ions. It encompasses an analysis of signal amplification strategies centered on engineered biorecognition elements and nanomaterials. The review also highlights recent advancements in ECL biosensing, encompassing dual-mode detection, engineered paper-based microfluidics, and the simultaneous detection of multiple ions. Furthermore, the article outlines the engineered future research trends and technological enhancements in ECL biosensing. The primary objective of this paper is to establish the theoretical framework for a compact, high-yield, and highly selective engineered ECL instant detector, enabling efficient, reliable, and sensitive monitoring of heavy metal ions.

**Table 1:** Heavy metals and health-related concentrations.

Heavy Metal	Maximum Acceptable Concentration (mg/L) (WHO)	Toxic Effects and Health Hazards	References
Lead (Pb)	0.01	Lead contamination can lead to nervous system damage, especially harmful to children’s brain development. It may also cause anemia, kidney damage, and digestive system problems.	[32]
Mercury (Hg)	0.001	Mercury poisoning can cause neurotoxicity, leading to brain and nervous system damage, reproductive problems, cardiovascular problems, and kidney damage. Mercury can enter the food chain and pose a risk to wildlife and humans.	[33]
Cadmium (Cd)	0.003	Poisoning may cause bone problems, kidney damage, and lung cancer. It is particularly harmful to children and may lead to mental impairment.	[34]
Chromium (Cr)	0.05	Chromium poisoning may cause respiratory problems, skin problems, and increase the risk of lung cancer.	[35]
Nickel (Ni)	0.02	Nickel poisoning may lead to allergies, heart and kidney disease, pulmonary fibrosis, lung cancer, and nasal cancer.	[36]
Copper (Cu)	2	Copper toxicity can lead to gastrointestinal problems, headaches, and liver damage; copper deficiency may lead to decreased immune system function and sluggish metabolism.	[37]
Zinc (Zn)	3	Zinc toxicity may cause gastrointestinal problems, poor healing, and even suppression of the immune system; zinc deficiency may lead to anemia, decreased immune system function, and growth retardation.	[38]
Iron (Fe)	0.3–1.0	Excessive iron intake may lead to gastrointestinal problems, while iron deficiency may lead to anemia.	[39]
Arsenic (As)	0.01	Long-term intake of high levels of arsenic may lead to skin damage, cardiovascular disease, lung cancer, and urinary problems.	[40]

## 2. Recent Progress of Engineered ECL Biosensors

Electrochemiluminescence (ECL) is a technique for generating light signals by electrochemical means. The ECL sensor consists of an electrochemical workstation and a photomultiplier [41]. It is based on a redox reaction in an electrochemical process that produces a detectable light signal under potential control through a chemiluminescent substance on the surface of the electrode. ECL typically entails the utilization of an electrolyte solution, an electrode material, and a luminescent marker to attain remarkably sensitive molecule detection.

In the past few years, there has been a notable surge in interest in the advancement of ECL biosensors. They are commonly used to detect and measure the concentration of proteins [42], nucleic acids, metabolites, and cancer markers [43] with high sensitivity and specificity [44]. The core of an engineered ECL biosensor is its biological recognition element, which usually uses high-specific biomolecules such as single/double-stranded DNA, aptamers, antibodies, and antigens to specifically interact with the molecules to be tested. An example is an engineered ECL nucleic acid sensor based on pyridine ruthenium polymer [Ru(bpy)_3_^2+^] labeling, whose detection mechanism is based on the recognition of specific nucleic acid targets [45]. First, the aptamer specifically binds to the target nucleic acid sequence, and then, under electrochemical excitation, the [Ru(bpy)_3_^2+^] marker produces an electrochemiluminescence signal. Its intensity is proportional to the concentration of the target nucleic acid, achieving highly sensitive and selective nucleic acid detection.

Compared with traditional optical or electrochemical detection methods, ECL biosensors have significant advantages, including excellent detection sensitivity and high biological specificity. For instance, in traditional fluorescence labeling techniques, background fluorescence often interferes with the signal, and ECL can greatly reduce background interference by detecting the time characteristics of the optical signal, such as luminescence at a specific potential, thereby improving the accuracy of the detection [46]. Additionally, ECL technology does not require an external light source, which simplifies the detection equipment and makes it easier to construct miniaturized instruments. Compared with electrochemical techniques, ECL also has excellent linear response range and high selectivity, so it is a powerful tool for biosensors and chemical analysis.

In the detection of heavy metal ions, engineered ECL biosensors usually use specific substances as recognition elements, combined with electrochemical signal converters, to convert biological signals acting on heavy metal ions into identifiable electrical signals [47]. Using electrochemiluminescence signal amplification to achieve extremely low concentration detection, through the selective recognition of biological recognition elements, the detection accuracy is improved. This approach has real-time monitoring capability and is suitable for online environmental monitoring. At the same time, combined with the application of miniaturization and nanotechnology, engineered ECL biosensors are developing towards the trend of diversification of detection methods, portability and real-time monitoring technology, and interdisciplinary cooperation, providing innovative solutions for environmental monitoring, biological research, and medical diagnosis [48].

## 3. Engineered ECL Biosensors on Metal Ion Detection

The core components of ECL biological systems can be divided into two categories: recognition elements and signal converters. The main function of the identification element is to detect the presence and concentration of the substance to be measured. The signal converter is responsible for converting the information sensed by the recognition element into observable and recorded signals, such as current intensity or ECL intensity. These two parts work together to form the key function of the ECL biological system. The signal amplification strategy, on the one hand, includes the efficient identification and signal transduction of the target, and, on the other hand, includes the amplification output of the transduction signal. Specifically, it includes signal amplification based on biological recognition elements and signal amplification based on nanomaterials.

### 3.1. Signal Amplification Strategy Based on Biometric Components

DNA biosensors utilize DNA as a biomolecular recognition element for the detection and measurement of specific molecules or analytical targets. Aptamers are highly specific molecules, usually oligonucleotides or their analogs, and DNA aptamer-based biosensors usually fall into a special category of DNA biosensors. They interact with the target molecule through a three-dimensional structure, similar to the matching of a lock and key. Due to this specific binding, aptamers are widely used for molecular recognition and capture in medical diagnostics, drug analysis, and environmental monitoring.

**Figure 1:**
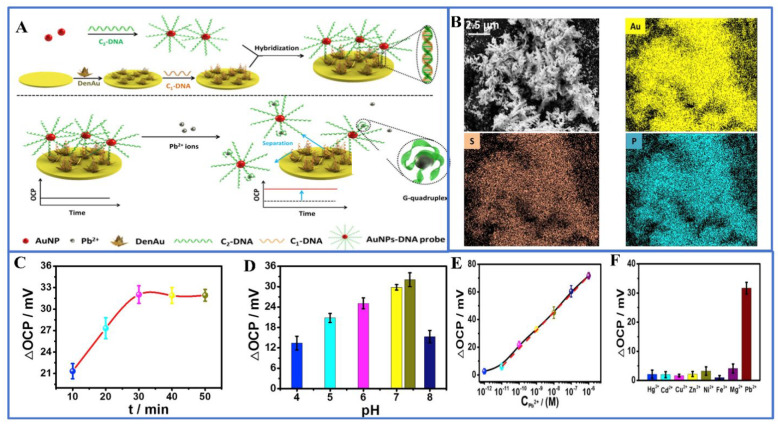
(**A**) Construction of aptamer sensor/DenAu/Au electrochemical biosensor platform; (**B**) SEM images of Aptasensor/DenAu and energy dispersive X-ray elemental maps of Au, S, and P elements; (**C**) detection time and (**D**) the effect of the pH value of the detection solution on the response of the sensor; (**E**) calibration curve of logarithm of Pb^2+^ concentration in PBS (pH 7.4) solution; and (**F**) selectivity of 1.0 μM interfering ions. Reprinted with permission from Ref. [50].

Upon binding with a specific metal ion, the aptamer can trigger the cleavage of the DNA strand it interacts with or the formation of a DNA-metal ion complex. This alteration in the DNA structure subsequently leads to changes in the ECL luminescence properties, enabling the detection of metal ions [49]. As everyone knows, the detection of Pb^2+^ depends on the specific base sequence in the aptamer (usually guanine-rich nucleotide G) that can form a stable G-quadruplex structure with Pb^2+^ [50] (Figure 1). The thymidine-rich nucleotide (T) sequence within the aptamer has the capacity to form a T-Hg^2+^-T complex with Hg^2+^. This interaction induces changes in the ECL signal, facilitating the detection of Hg^2+^. For the detection of Cd^2+^, non-repetitive ssDNA sequences rich in thymidine (T) and guanine (G) are employed to capture Cd^2+^. Building upon this principle, Liu et al. successfully designed an aptamer electrochemical biosensor utilizing the thymine-Hg^2+^-thymine (T-Hg^2+^-T) structure for the assessment of mercury ions in tap water samples [51]. Hg^2+^ recognition by the aptamer leads to the formation of a hairpin structure, resulting in an electrochemical signal near the sensor’s surface and ultimately generating an electrochemical response. Experimental outcomes demonstrate that the sensor exhibits a strong linear correlation within the concentration range of mercury ions from 0.01 to 500 nM (R^2^ = 0.9994), with an impressively low detection limit of 0.005 nM. Meanwhile, Lin et al. utilized G-quadruplexes based on iridium (III) complexes for As (III) determination [52]. The principle of this method is that the G-rich sequence is initially combined with the As (III) aptamer in the hairpin structure of the aptamer region by introducing a DNA sequence with two loops, including the As (III) aptamer sequence. Upon the introduction of As (III), the formation of the As–aptamer complex leads to the release of the complementary DNA sequence. Subsequently, the released G-quadruplex sequence adopts a G-quadruplex structure, which is selectively recognized by the iridium (III) complex, resulting in luminescence enhancement signals. This method offers a remarkable sensitivity, with a minimum detectable concentration as low as 7.6 nM.

The programmability of DNA molecules endows them with diverse self-assembly capabilities to form different structures. This feature allows us to accurately control the DNA circuit through target induction to achieve signal amplification, thereby improving the performance of the aptamer biosensor. At the same time, it is always associated with hybridization chain reaction (HCR), rolling circle amplification (RCA), entropy-driven catalysis (EDC), and clustered regularly interspaced short palindromic repeats (CRISPR) technology [53] to enhance the detection signal and thereby obtain clear and accurate detection results. By designing aptamers that specifically recognize the target analyte, the biosensor can selectively capture and detect the required metal ions in the presence of interfering substances. In this paper, several advanced DNA signal amplification strategies for metal ion detection will be presented.

#### 3.1.1. HCR Amplification Strategy

The hybridization chain reaction (HCR) is a DNA self-assembly process used to amplify signals from target molecules. Typically, HCR contains two or more DNA hairpins. When the target molecule is present, these trigger DNA hairpins will start alternately and self-assemble one after another to form an incision double-stranded DNA nanostructure containing a large number of repeating units, thereby achieving a significant enhancement of the signal for detection of the target molecule [54].

**Figure 2:**
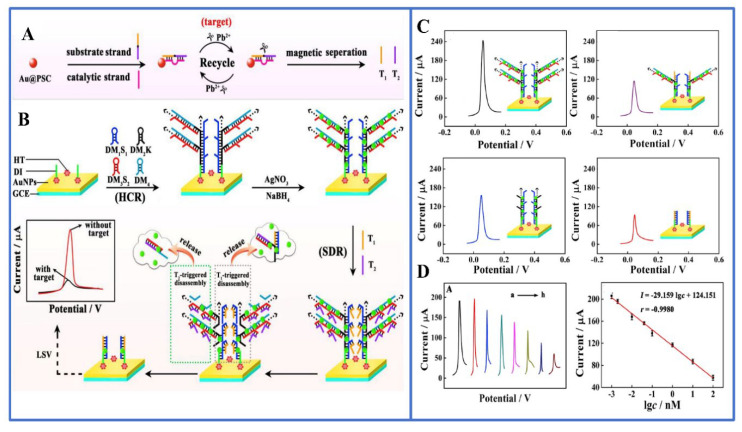
(**A**) Synthesis of DNA triggers (T_1_ and T_2_); (**B**) trigger-induced decomposition of branched DNA polymers for ultrasensitive determination of Pb^2+^; (**C**) linear sweep voltammetry reactions of four different dissociation methods; (**D**) biosensor detection of Pb^2+^ series concentrations; error lines indicate the standard deviation of three individual determinations (n = 3). Reprinted with permission from Ref. [57].

The HCR reaction possesses significant advantages. HCR functions under mild conditions, eliminating the necessity for costly equipment or intricate procedures. Its straightforward operating principle makes it highly adaptable for integration with other reactions or substances, while also being less susceptible to signal interference in reporting methods. Additionally, HCR is an enzyme-free amplification technique. The DNA self-polymerization process, initiated by the initiator, results in the formation of an elongated double-stranded DNA polymer. This facilitates the embedding of a greater quantity of ECL reagents, such as [Ru(phen)_3_]^2+^, into the double-stranded DNA or enhances the binding of DNA labeled with ECL reagents. Consequently, this process leads to a substantial enhancement in electrochemiluminescence (ECL) [55]. For instance, Wang and his team have created an electrochemiluminescence aptamer sensor using HCR and DNA-QD nanostructures. The sensor uses an HCR amplification strategy to create a new DNA-QD nanostructure by combining cadmium sulfide (CdS) quantum dots with streptavidin. This structure enables highly specific recognition of Cd^2+^ and significantly enhances the ECL signal [56]. Xie and colleagues developed a label-free biosensor for the highly sensitive electrochemical detection of lead ions (Pb^2+^) [57]. This sensor relies on the effective decomposition of branched DNA polymers and repetitive amplification of target migration. As shown in Figure 2, to begin, they immobilize the branched DNA polymer created through HCR on the electrode, which serves as a carrier enriched with silver nanoparticles (Ag NPs), generating a robust initial current signal. Subsequently, the molecular triggers T_1_ and T_2_, produced through the target migration cycle amplification, break down the branched DNA polymer, leading to a substantial reduction in the current. This process enables highly sensitive detection of Pb^2+^. In comparison to conventional approaches, this method exhibits superior decomposition efficiency. Experimental results demonstrate that the detection limit for Pb^2+^ with this biosensor has been lowered to an impressive 0.24 pM, showcasing outstanding performance.

#### 3.1.2. RCA Amplification Strategy

**Figure 3:**
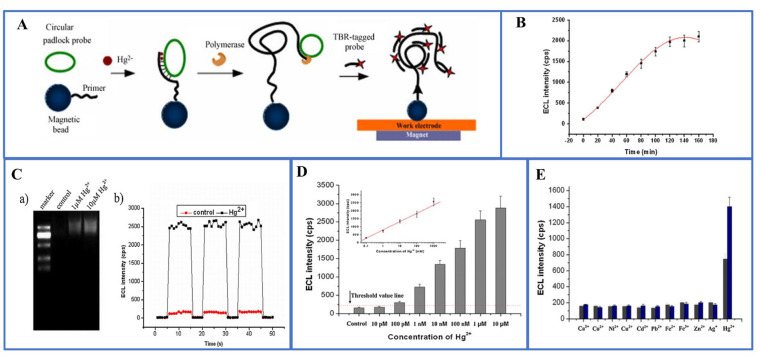
(**A**) Schematic diagram of a magnetic bead-based Hg^2+^ mediated rolled-ring amplification-electrochemiluminescence sensor for Hg^2+^ detection in aqueous solution; (**B**) the curve of ECL intensity of Hg^2+^ with time; (**C**) the verification of RCA product in 1% agarose gel electrophoresis, and the ECL intensity corresponding to 1 μM Hg^2+^ (no target control); (**D**) a schematic diagram of the sensitivity of the sensor to detect Hg^2+^. Illustration: calibration curve of 0.1 to 1000 nM Hg^2+^. (**E**) Selectivity test with all competing metal ions tested at 1 μM (gray) and 100 μM (blue). Reprinted with permission from Ref. [59].

RCA is an excellent nucleic acid amplification strategy. The mild isothermal reaction conditions of HCR provide excellent stability and efficiency, even in complex environments. This method necessitates only a small quantity of circular DNA template and employs phi29 DNA polymerase to catalyze the extension and replication of the DNA chain, leading to an expansion of the complementary strand of the DNA template by hundreds of times. The core principle of RCA involves a circular template that guides the generation of multiple repetitive sequences, leading to the synthesis of ultra-long single-stranded DNA, and the enhancement of the ECL signal based on its loaded RCA product or based on the rich in situ form in the ECL luminophore. For example, Pang et al. developed a highly sensitive biosensor for the quantitative detection of Pb^2+^ [58]. This biosensor employed a unique combination of RCA and hemin amplification strategies. Under optimized conditions, the biosensor achieved an impressively low detection limit of 3.3 pM, with a linear range spanning from 10 pM to 1000 nM. Zhou et al. developed a novel Hg^2+^ ECL sensor strategy by specifically recognizing and reacting to T-Hg^2+^-T biomimetic structures using Bst DNA polymerase [59]. In this approach (Figure 3), they designed primers labeled with magnetic beads that are complementary to the circular padlock probe region. However, these primers were intentionally created with two T-T mismatches at the 3′ end. Bst DNA polymerase was unable to extend the mismatch primers when no Hg^2+^ was present. However, once Hg^2+^ is present, a stable T-Hg^2+^-T structure can be formed, which induces the DNA polymerase to employ the RCA mechanism for extension and amplification reactions. Subsequently, the resulting RCA product hybridizes to a tris(bipyridyl)ruthenium (TBR)-labeled probe and is detected by an ECL platform. Currently, this method has demonstrated a sub-nanomolar level detection limit and excellent selectivity for the detection of a wide range of interfering metal ions in the assay.

#### 3.1.3. EDC Amplification Strategy

**Figure 4:**
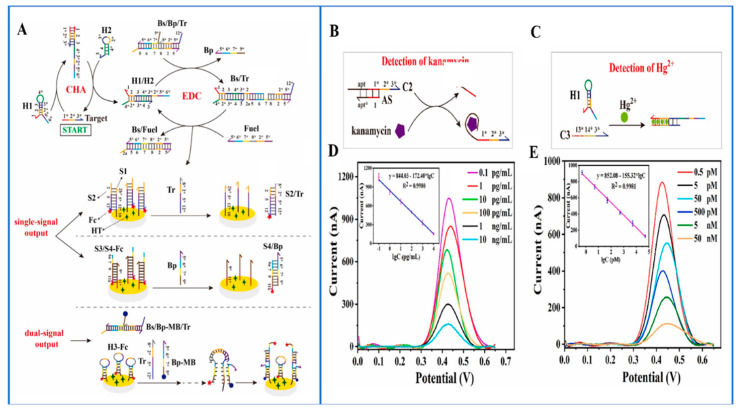
(**A**) A versatile electrochemical biosensor platform using the CHA-EDC cascade amplification circuit; (**B**) kanamycin and (**C**) Hg^2+^ activation within the CHA–EDC circuit (the logarithm of concentration between 0.1 pg/mL and 10 ng/mL); (**D**) electrochemical responses to varying kanamycin concentrations and (**E**) to Hg^2+^ (the range of 0.5 pM-50 nM). Reprinted with permission from Ref. [62].

DNA engineering is distinguished by its molecular programmability and design flexibility. When integrated with the entropy-driven process of DNA hybridization, it allows for the construction of enzyme-free signal cascade amplification circuits and sensors. The driving force behind this approach stems from the natural tendency of the reaction system to undergo spontaneous amplification without the need for costly enzymes and antibodies. The entropy-driven ECL biosensor uses the entropy change caused by the combination of metal ions and aptamers to detect the presence and concentration of metal ions, and achieves highly sensitive metal ion detection by monitoring the change of the luminescence signal. For example, Yu’s research team developed an electrochemiluminescence biosensor that relies on the entropy-driven strand displacement reaction (ETSD) and tetrahedral DNA nanostructure (TDN) to achieve signal amplification. This innovative approach was used for the detection of biomarkers associated with acute myocardial infarction, demonstrating outstanding specificity [60]. Kim and colleagues designed a DNA nanomachine capable of converting protein signals into nucleic acid output, enabling real-time analysis and swift response detection in whole blood and plasma samples [61]. In addition, Zhu and colleagues ingeniously combined a potent Catalytic Hairpin Assembly (CHA) with a cascade amplification system and an electrochemical analysis technique to engineer a highly sensitive biosensor platform utilizing DNA as the detection model. This engineered platform successfully enabled the sensitive detection of aptamer substrates (kanamycin) and certain metal ions (Hg^2+^) [62]. As shown in Figure 4, this approach represents an innovative engineering paradigm for creating cascade systems, leveraging isothermal, enzyme-free signal amplification technology. It introduces a pioneering strategy for designing highly efficient, versatile, and environmentally friendly cascade reaction circuits. The detection methodology involves harnessing target DNA as the trigger for Catalytic Hairpin Assembly (CHA) and encoding the catalytic sequence of exonuclease III (EDC) within the exposed sequence of the CHA product double-stranded DNA (dsDNA). This leads to the development of a target-triggered CHA–EDC cascade system. The products of this cascade circuit can engage in a chain displacement reaction with the signaling probe on the electrode surface, ensuring the highly sensitive detection of the target. Furthermore, an engineered proportional biosensor platform was established by capitalizing on the by-product chain generated in the EDC reaction, thereby enhancing the detection reproducibility and system stability.

In general, these DNA amplification strategies have brought significant breakthroughs in the field of metal ion detection, making up for the shortcomings of traditional detection methods in terms of sensitivity and specificity. The innovation of these strategies is that they all use DNA molecules as the basis for detection, fully utilizing the self-assembly and complementarity of DNA. Not only that, they have excellent metal ion specificity, allowing highly selective detection in complex sample matrices. DNA-based sensing technologies also show great potential in practical applications. For example, in the environment and in the medical field, they can be used to monitor heavy metals, and thereby detect and solve problems in time [63]. In addition, they provide powerful tools in the fields of food safety, pharmaceutical analysis [64], and materials science [65].

#### 3.1.4. CRISPR/Cas Amplification Strategy

Lately, CRISPR, along with its companion Cas protease, has emerged as a compelling choice for precise and effective genome editing [66,67]. Furthermore, modified versions such as Cas12, Cas13 [68,69], and Cas14 [70] have garnered attention due to their remarkable ability to execute side-branch cleavage, allowing for the precise and efficient cutting of RNA or DNA molecules. When the Cas protein/guide RNA complex identifies a target nucleic acid, it can cleave single-stranded reporter genes without the need for specific incisions. Cas12 and Cas14 are primarily engineered for DNA targeting, whereas Cas13 is specifically designed for RNA targeting [71]. A diverse range of CRISPR-based assays has been formulated through the integration of nucleic acid amplification techniques such as PCR and RCA. These methodologies harness the power of the CRISPR/Cas system, delivering swift response times, user-friendly operation, remarkable specificity, and heightened sensitivity. Crucially, they do not hinge on costly laboratory apparatus, establishing them as a celebrated advancement in the domain of molecular detection technology.

**Figure 5:**
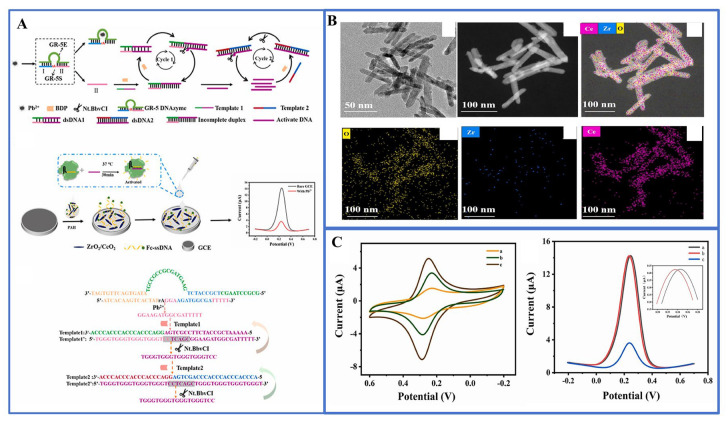
(**A**) Schematic diagram of the SM-E-CRISPR biosensor for the detection of lead ions, and the detailed workflow of DNA sequences in the SDA cycle; (**B**) TEM image and elemental mapping diagram of ZrO_2_/CeO_2_ nanorods; (**C**) CV response of different modified electrodes, DPV response after incubation without and with Pb^2+^. Reprinted with permission from Ref [73].

For example, the lateral flow assay using CRISPR-Cas12 is employed for the rapid (less than 12 min), accurate detection of the SARS-CoV-2 virus in respiratory swab RNA extracts [72]. Taking advantage of the unique combination of Cas12a and electrochemiluminescence (ECL) technology, Liu et al. proposed a novel electrochemical luminescence biosensing platform based on Cas12a for the detection of human papillomavirus subtype (HPV-16) DNA without the need for target amplification [73]. Cas12a utilizes its dual-recognizing mechanism to enhance specificity and cis-cleavage capability, resulting in signal amplification, with a detection limit of 0.48 pM. Similarly, DNAzymes, which are catalytically active DNA molecules, possess the capability to selectively bind to metal ions and convert metal ion recognition events into nucleic acid sequence data. Consequently, the fusion of the CRISPR/Cas system with DNAzymes holds the potential to streamline the detection of metal ions. Li and colleagues harnessed precise signal transduction involving aptamers and DNAzymes to identify sodium ions (Na) within the CRISPR/Cas12a biological framework. This innovative approach yielded highly sensitive and remarkably specific Na^+^ detection, achieving detection thresholds as low as 4.3 nM. Yue et al. have pioneered the development of an electrochemical biosensor known as E-CRISPR, which is based on CRISPR/Cas12a technology, demonstrating remarkable specificity in detecting Pb^2+^ (as depicted in Figure 5). In this approach, the GR-5 DNAzyme plays an intermediary role, facilitating the conversion of Pb^2+^ nucleic acid signals into single-stranded DNA, thereby initiating strand displacement amplification (SDA) reactions. The exceptional sensitivity achieved in this method can be attributed to a triple-cycle amplification process, including the dual cycle formed by SDA itself and the inherent signal-enhancing properties of CRISPR/Cas12a. As a result, the SM-E-CRISPR detection platform achieves a sensitivity level in the picomolar (pM) range [74]. This provides a straightforward, highly sensitive, and real-time method for detecting various other heavy metal ions.

### 3.2. Signal Amplification Strategy Based on Nanomaterials

The rapid development and improvement of nanotechnology provide a wide range of possibilities for the assembly of biosensors. Nanomaterials play an important role in the composition of ECL biosensors [75,76]. On the one hand, the unique surface effect and quantum effect of nanomaterials give them significant luminescence intensity and excellent stability. They have gradually replaced traditional luminescent markers (such as luminol and [Ru(bpy)_3_]^2+^) and become the first choice of luminescent materials for a new generation of biosensors [77]. On the other hand, the microscopic tunability of nanomaterials enables them to change the chemical state of the electrode surface at the molecular level, increasing key parameters such as specific surface area, number of active sites, and conductivity, thereby enhancing the signal amplification of the ECL sensor. Therefore, the signal amplification strategy of ECL systems based on nanomaterials is usually a special modification of the working electrode surface. The generation efficiency of ECL signals can be improved by introducing efficient ECL luminescent groups (such as gold nanoparticles or organic small molecule markers), thereby further improving the detection sensitivity. Furthermore, by using the surface enhancement effect of nanomaterials, the biorecognition elements are fixed on the surface of nanostructures, which can improve the detection sensitivity and selectivity of ECL signals.

#### 3.2.1. Quantum Dots

Quantum dots (QDs), semiconductor nanocrystals, typically consist of compounds from II-VI, III-V, and IV-VI group elements [78]. In contrast to traditional ECL reagents such as bipyridine ruthenium and luminal, quantum dots employed as ECL reagents offer distinctive features: (1) their emission wavelength can be precisely tuned by adjusting their size; (2) quantum dots exhibit broad absorption spectra across various energy levels; (3) they provide high luminescence efficiency and demonstrate robust stability; (4) they can serve as both donors and acceptors in luminescence resonance energy transfer (RET) processes, enhancing their versatility in applications; and (5) they have strong aptamer adsorption ability. In the ECL sensor, quantum dots act as emitters, and when the target metal ions bind to the biorecognition element, they induce an electrochemiluminescence process. Through electrochemical excitation, the quantum dots enter the excited state and release energy when returning to the ground state to generate a luminescence signal. This method has high sensitivity and accuracy, and is suitable for environmental monitoring, biomedical research, and other fields [79].

**Figure 6:**
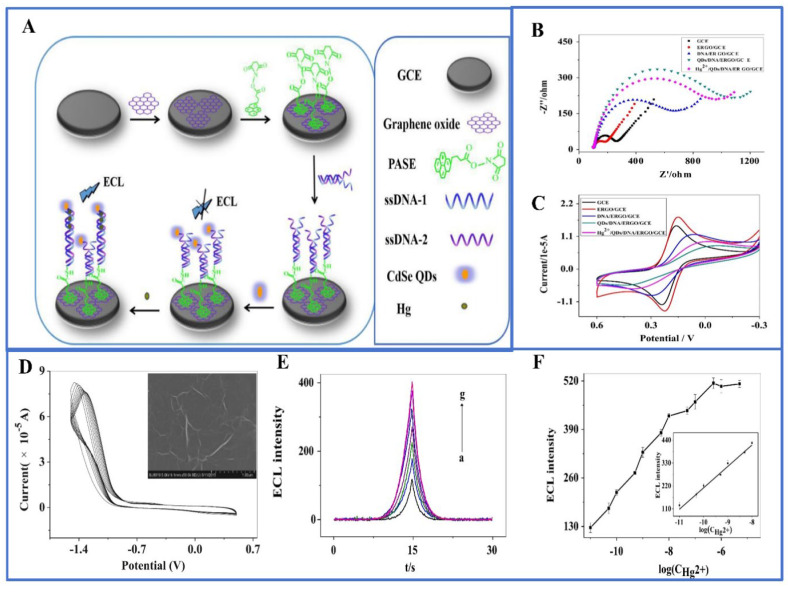
(**A**) DNA ECL biosensor and its detection of Hg^2+^; (**B**) EIS and (**C**) CV measurements; (**D**) CV measurement of GO; inset is an SEM image of ERGO; (**E**) ECL intensity of different concentrations of mercury (the measurement of Hg^2+^ was conducted on an MPI-A ECL analyzer with a potential range of −1.5~0 V at a scan rate of 100 mV/s); (**F**) the relationship between ECL intensity of the sensor and the concentration of mercury. Reprinted with permission from Ref [84].

**Figure 7:**
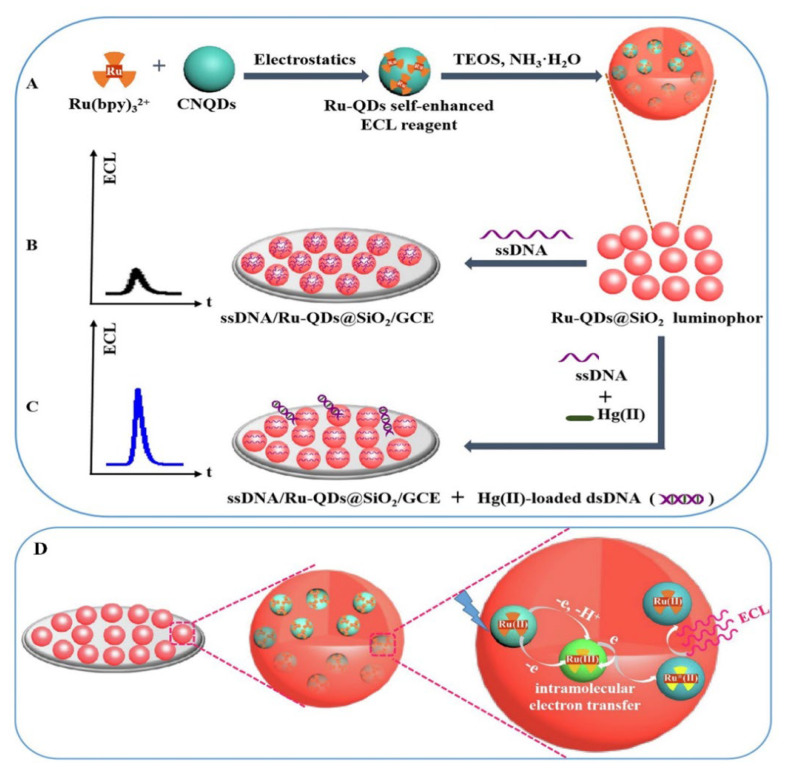
(**A**) Synthesis of the Ru-QDs self-enhanced ECL reagent and Ru-QDs@SiO_2_ ECL material; (**B**) construction of label-free ECL biosensor; (**C**) Hg^2+^ was detected by the developed sensing strategy; (**D**) an illustrative diagram depicting the self-enhanced ECL process occurring within SiO_2_ nanoparticles. Reprinted with permission from Ref [85].

A variety of quantum dots are widely employed in ECL sensors, including sulfur metalized quantum dots, carbon nanodots (CQDs) [80], graphene quantum dots (GQDs), silicon quantum dots [81], carbon nitride quantum dots (CNQDs), and doped quantum dots [82]. They can serve as luminophores, co-reactants, or donors/acceptors for energy transfer for the detection of metal ions. For example, graphene quantum dots (GQDs), as a zero-dimensional graphene structures, exhibit excellent luminescence properties. In this context, Anusuya and his research team designed an ECL aptasensor using high-brightness GQDs as luminescent materials for extremely sensitive detection of Hg^2+^, Cd^2+^, and Pb^2+^ [83]. Li et al. built a highly sensitive ECL sensor for detecting trace amounts of Hg^2+^ in water by combining CdSe quantum dots, graphene oxide, and double-stranded DNA (Figure 6) [84]. Although quantum dots have potential applications in biological systems, there are inevitably some problems such as cytotoxicity and instability. To address these challenges, Li et al. utilized SiO_2_ nanoparticles (NPs) to encapsulate carbon nitride quantum dots (CNQDs), enhancing their stability and minimizing external environmental interference. CNQDs are known for their low toxicity, excellent biocompatibility, and stability. As shown in Figure 7, Li et al. introduced a novel self-enhanced electrochemiluminescence (ECL) emitter (Ru-QDs) by combining [Ru(bpy)_3_]^2+^ with CNQDs [85]. Furthermore, they encapsulated Ru-QDs within SiO_2_ nanoparticles, successfully establishing a label-free self-enhanced ECL biosensor approach for highly sensitive and selective detection of Hg^2+^. This was achieved by leveraging the differential adsorption capacity of Ru-QDs@SiO_2_ nanoparticles for single-stranded DNA (ssDNA) containing thymidine (T) and double-stranded DNA (dsDNA) induced by Hg^2+^. This method allows for the measurement of Hg^2+^ in the concentration range of 0.1 nM to 10 μM, with an impressively low detection limit of 33 pM.

#### 3.2.2. Metal Nanoclusters

In general, the sensitivity of ECL biosensors to detect metal ions is improved mainly through the following two strategies: (1) increasing the load of signal tags or recognition elements using nanomaterials with high specific surface area; and (2) the introduction of nanomaterials with catalytic activity to enhance the signal. Commonly used nanomaterials include metal nanomaterials, carbon-based nanomaterials, and magnetic nanomaterial. The unique properties of these materials make them widely used in many fields such as environmental protection and biological health monitoring.

Metal nanoclusters are micro-nano clusters composed of a few to hundreds of metal atoms [86]. They usually have special electronic structures and optical properties, which are different from those of large-sized metal particles or bulk materials. Metal nanoclusters are highly regarded for their unique electrical, magnetic, and optical properties. Therefore, by adjusting the size and shape of Au NPs, different biosensing requirements can be met. For example, Dong et al. studied the effect of different sizes (6, 16, 25, 38, 68, and 87 nm) of gold NPs on the ECL of luminol, and found that the 16 nm Au NP-modified electrode had the greatest catalytic activity. Recently, Yuan and colleagues engineered an electrochemiluminescence resonance energy transfer (ECL-RET) system to achieve adaptive and proportional detection of Pb^2+^. This innovative system involved the application of gold nanoparticles functionalized with fullerene nanocomposites (AuNPs@nano-C60) onto a glassy carbon electrode (GCE). The detection limit of the ECL sensing platform is 3.5 × 10^−13^ M. Moreover, Zhou and collaborators devised an exceptionally sensitive and selective electrochemical biosensor tailored for the detection of lead ions (Pb^2+^) in environmental water samples [87]. The sensor’s operational concept relies on the cooperative catalytic action of porous Au-Pd bimetallic nanoparticles and DNA enzymes. In their investigation, Au-Pd bimetallic nanoparticles were employed to enhance the surface coverage of nanostructures, consequently boosting the labeling density of G-DNA molecules. The experimental outcomes demonstrated the exceptional Pb^2+^ detection capability of the sensing platform across a range from 1.0 pM to 100 nM, with a low detection limit (LOD) of 0.34 pM.

#### 3.2.3. Carbon-Based Nanomaterials

Carbon-based nanomaterials, including carbon nanotubes and graphene, stand out as optimal options for both heavy metal ion detection and removal. This is attributed to their outstanding electrical conductivity and chemical reactivity [88,89]. As an illustration, Tang et al. crafted DNA-S1-modified poly(5-formylindole)/reduced graphene oxide nanocomposites to attain remarkably selective detection of Hg^2+^. In spite of the use of nanomaterials, suitable sensing strategies are crucial for performance enhancement. ECL resonance energy transfer (ECL-RET), which emphasizes the overlap between the ECL spectrum of the donor and the absorption spectrum of the acceptor, is a promising strategy [90].

**Figure 8:**
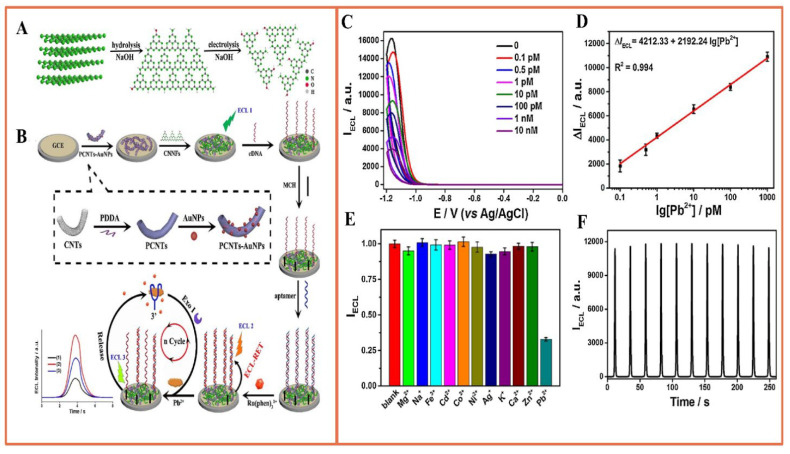
(**A**) Two-step preparation strategy of CNNF (**B**) and the fabricated Pb^2+^ aptamer sensor; (**C**) ECL response of the sensor in the existence of different concentrations of CEA; (**D**) calibration curve for Pb^2+^ determination; (**E**) selectivity of the sensor for Pb^2+^ (the ΔIECL value was compared in the presence of Pb^2+^ and ten other different metal ions, all at the same concentration of 1.0 nM); (**F**) stability test of the sensor in the presence of 1 nM Pb^2+^. Reprinted with permission from Ref [91].

Graphitic carbon nitride (CN), consisting of sp2 bonded carbon and nitrogen, has important potential for applications in photocatalysis and electrocatalysis, etc. Peng et al. designed a sensor for ultra-sensitive lead detection through ECL-RET involving graphitic carbon nitride nanofibers (CNNFs) and Ru(phen)_3_^2+^ [91]. Utilizing the amplification and stabilization of carbon nanotubes and gold nanoparticles, ECL-RET underwent triple amplification from CNNFs to Ru(phen)_3_^2+^ (Figure 8), as well as nucleic acid exonuclease-assisted target recovery for detection. These combined amplification approaches yielded an exceptionally sensitive aptamer sensor for Pb determination, achieving an impressively low detection limit of 0.04 pM. The probe was successfully applied to analyze environmental samples, producing satisfactory results.

#### 3.2.4. Porous Nanomaterials

In addition, porous nanomaterials, such as mesoporous silica, possess unique properties such as extended π-conjugated backbones, large surface areas, and specialized porous structures [92]. Zhang et al. prepared Fe_3_O_4_ @SiO_2_ composites via core-shell technology, in which superparamagnetic Fe_3_O_4_ nanoparticles are the core [93]. The silica layer used to capture heavy metal ions was synthesized by the sol–gel method. The proposed analysis method can be used to detect a variety of heavy metal ions in milk with high sensitivity.

Metal-organic framework (MOF) is a composite material self-assembled by organic ligands and metal ions through coordination bonds, with an internal microporous structure. It can take advantage of the electrochemiluminescence activity of the organic ligand itself, and can also be combined with the electrochemiluminescence active substance to enhance its luminescence performance [94]. Liu and his group synthesized Ag-MOF as an electrochemiluminescence luminophore for the detection of mercury ions in water samples [95]. To improve the ECL signal stability, chitosan (CS) and gold nanoparticles (Au NPs) were introduced for modification. A biosensor for the specific detection of mercury ions was established by constructing mercury ion aptamers and the spatial site-blocking effect of streptavidin. Upon the introduction of mercury ions, the nucleic acid aptamer detached from the electrode surface, causing a decrease in the quantity of streptavidin. This led to a gradual increase in the intensity of the electrochemiluminescence (ECL) signal. Consequently, the concentration of mercury ions in the sample can be precisely measured based on the variations in ECL signal intensity.

One of the future development directions of ECL is to develop new near-infrared (NIR) luminescent materials that are efficient and free from interference, which will lead to major breakthroughs and innovations in the field of analysis [96]. Because the interference in the NIR region is relatively small, this creates important opportunities for improving contrast, signal-to-noise ratio, and ECL efficiency. Porphyrins are renowned for their outstanding characteristics, including high absorbance of visible light, a tunable band gap, and favorable optical and redox properties. Furthermore, the majority of porphyrins exhibit deep-penetrating red or near-infrared fluorescence (FL) emissions [97]. In Yu’s research, as an illustration, they effectively created luminescent MOFs based on porphyrins (pMOFs) and achieved a consistent improvement in electrochemiluminescence (ECL) signals [98]. In addition, inspired by the excellent performance and quantum size advantages of porphyrin materials, Zhang’s team successfully prepared three kinds of extremely small and uniform porphyrin dots using the exfoliation method, which are TCPP dots, ZnTCPP dots, and pMOF dots [99]. All of them demonstrate consistent and potent near-infrared ECL capabilities. By leveraging the advancement of tetra(4-carboxyphenyl) porphyrin (TCPP) dots, in conjunction with lead-dependent DNAzyme and hybridization chain reaction amplification, a label-free ECL biosensor was designed for the detection of trace amounts of lead. The outcomes revealed an exceptionally low detection limit for Pb^2+^ at 1.2 picomoles per liter (p mol·L^−1^) within a range spanning from 10 pmoL·L^−1^ to 1 μmol·L^−1^. In addition, compared with the standard system of Ru(bpy)_3_^2+^, the three porphyrin points in S_2_O_8_^2−^ increased the ECL efficiency by 56, 44, and 39 times in the cathode system, respectively. This research offers novel design and synthesis approaches for ECL luminescent materials with high performance, while also laying the foundation for the creation of ECL sensors characterized by straightforward fabrication, minimal background signals, and exceptional sensitivity.

## 4. New Technologies of Engineered ECL Biosensors

Although the current ECL sensors have shown satisfactory selectivity and sensitivity in the detection of metal ions, they are usually only suitable for laboratory environments and struggle to meet the actual needs of on-site analysis and environmental monitoring. Therefore, in order to overcome these limitations, this section introduces a series of novel methods, such as innovative solutions that rely on intelligent systems and microfluidic technology, including dual-modal detection based on the ECL method combined with other methods, portable paper-based detection, and simultaneous detection of multiple ions [100,101]. These new methods are expected to achieve more convenient, rapid, and multifunctional metal ion analysis to meet the urgent needs of environmental and health monitoring.

### 4.1. Dual-Mode Analysis

**Figure 9:**
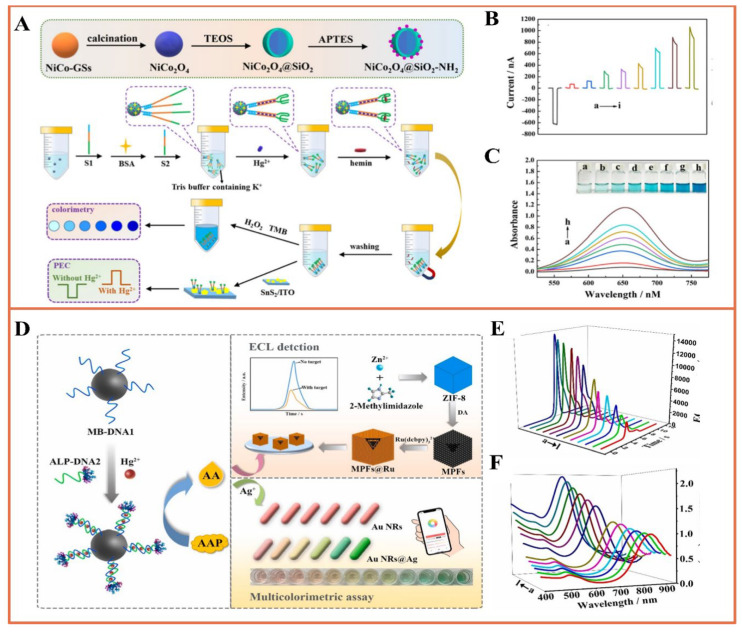
(**A**) Preparation of NiCo_2_O_4_@SiO_2_-NH_2_@S1-S2 and schematic diagram of colorimetric and PEC dual-modal detection of Hg^2+^; (**B**) photocurrent response of different concentrations of Hg^2+^ (from a–i: 0~500,000 pM); (**C**) UV-visible spectra of different concentrations of Hg^2+^ of UV-Vis spectra (a–h: 0~10,000 pM) [104]; (**D**) preparation of bimodal ECL and multicolorimetric assay based on ECL response; (**E**) ECL response and (**F**) UV-Vis spectral response of different concentrations of Hg^2+^ (a–l: 0~500 nmol L^−1^). Reprinted with permission from Ref [105].

It is well known that ECL technology, as a powerful analytical tool, has the characteristics of high sensitivity, excellent controllability, and low background. Therefore, the accuracy of monitoring can be effectively improved when a dual-modal analysis platform for metal ions is constructed based on ECL sensing [92,102]. Thanks to its integrated cross-reference correction feature, it has the capability to minimize the occurrence of false positive outcomes when dealing with samples containing intricate components in challenging environments. For example, Hao et al. proposed a dual-mode aptamer sensor based on a multi-functional multi-in-one probe (Fe_3_O_4_@Au-ssDNA&Ru-NH_2_) for ECL and fast scanning voltammetry (FSV), which achieved highly selective detection of sub-nanomolar Pb^2+^ [103]. Fu and colleagues devised a dual-mode sensing platform for Hg^2+^ detection that harnesses the remarkable catalytic activity of 3,3′,5,5′-tetramethylbenzidine (TMB) and the robust photocurrent polarity conversion property of the split G-quadruplex-heme complex on the SnS_2_ photoanode. This platform features the assistance of magnetic NiCo_2_O_4_@SiO_2_-NH_2_ spheres and supports both colorimetric and photoelectrochemical (PEC) detection [104]. As shown in Figure 9A–C, the LOD based on colorimetric determination was 3.8 pM. At the same time, the LOD of photoelectrochemical determination based on the superior photocurrent polarity of heme was 2.3 pM. In this regard, Ma et al. developed a novel ECL and colorimetric dual-modal analysis platform for portable and visual analysis of Hg^2+^ in water samples based on smartphones (Figure 9D–F) [105]. In order to achieve this goal, first, a thymine (T)-rich DNA probe was used to capture Hg^2+^, in which the DNA probe was ligated to magnetic beads (MBs-DNA) and alkaline phosphatase-labeled DNA (ALP-DNA2) to form a T-Hg^2+^-T structure complex. Subsequently, following magnetic separation, alkaline phosphatase (ALP) initiated the hydrolysis of ascorbic acid-2-phosphatase (AAP) that was added, generating ascorbic acid (AA). AA could react with Ru(dubpy)_3_^2+^ and co-reactant S_2_O_8_^2−^, causing a reduction in the cathodic electrochemiluminescence (ECL) emission signal on the surface of the glassy carbon electrode (GCE), enabling the ECL detection of mercury. In addition, AA could also reduce silver nitrate and deposit it onto gold nanorods, resulting in a blue shift in the localized surface plasmon resonance (LSPR) peak of the gold nanorods. This color change allowed the visual colorimetric determination of mercury. Under the optimized conditions, the LODs of the dual-mode mercury determination were 0.32 pM and 0.57 pM, respectively, which were higher than those of the single method. Significantly, the addition of smartphones significantly cuts inspection costs, simplifies the process, and enables rapid on-site testing without the need for additional equipment.

### 4.2. Paper-Based Microfluidic Detection

The combination of microfluidics and smartphones for immediate detection (POCT) has emerged as a rapid and convenient detection method, enabling highly integrated and intelligent detection systems with the advantages of low reagent consumption, high automation, and miniaturization [106,107]. These new ways are expected to enable faster and more efficient analysis of metal ions to meet the needs of environmental monitoring and practical applications. Ma’s research group designed a portable microfluidic sensing platform that can be controlled via a smartphone and relies on thermal capillary convection for the detection of the hazardous substance Pb^2+^ [108]. It exhibited a wide range of 0.01 μg/L~2100 μg/L and a low detection limit of 0.005 μg/L when analyzing river water, fish, and human serum samples.

**Figure 10:**
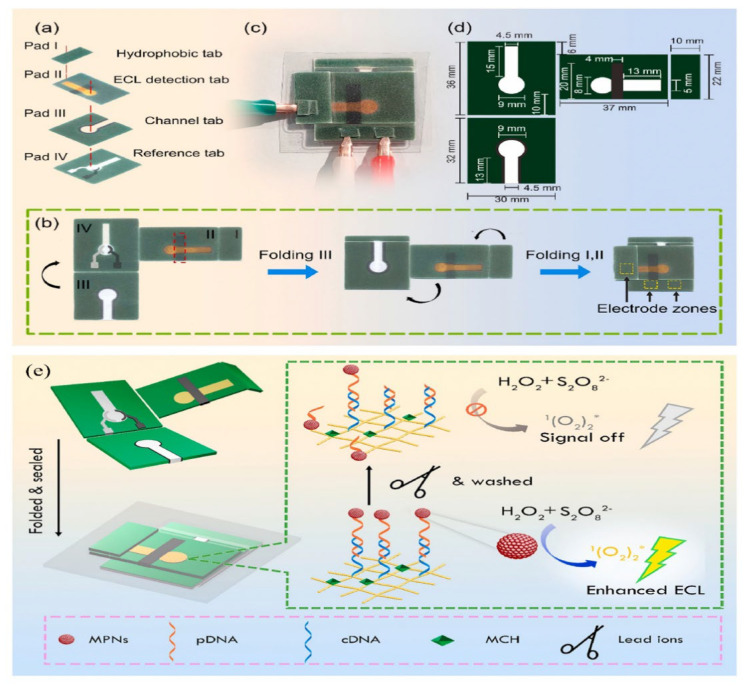
Design and manufacture of laboratory equipment on fully sealed paper: (**a**) image showing the wax pattern on the integrated paper-based platform after the baking process; (**b**) folding process of the LOP ECL device and (**c**) assembly process; (**d**) assembly diagram; (**e**) the schematic diagram of fully sealed ECL paper-based platform for Pb^2+^ detection. Reprinted with permission from Ref [109].

Laboratory-on-Paper (LOP) is of great interest for immediate diagnosis and environmental testing. Inspired by this, Zhu et al. developed a paper-based ECL Pb^2+^ detection platform by introducing plastic encapsulation technology into LOP devices [109]. As shown in Figure 10, oligonucleotide probes functionalized with red myristoylated Pt nanomaterials (MPNs) were used for electrode modification, and the combination of paper electrodes with functional regions separated by multilayers of waxed paper enhanced the utility of rapid detection. The integrated platform achieves a wide linear range of 0.01 nM to 0.05 μM, with Pb^2+^ detection down to a detection limit of 0.004 nM. In summary, the on-site ECL sensing platform based on paper is known for its reliability, stability, and accuracy when monitoring heavy metal ions in various matrices, particularly in environmental applications. This is attributed to its ease of use, portability, straightforward operation, and cost efficiency.

### 4.3. Detection of Various Metal Ions

**Figure 11:**
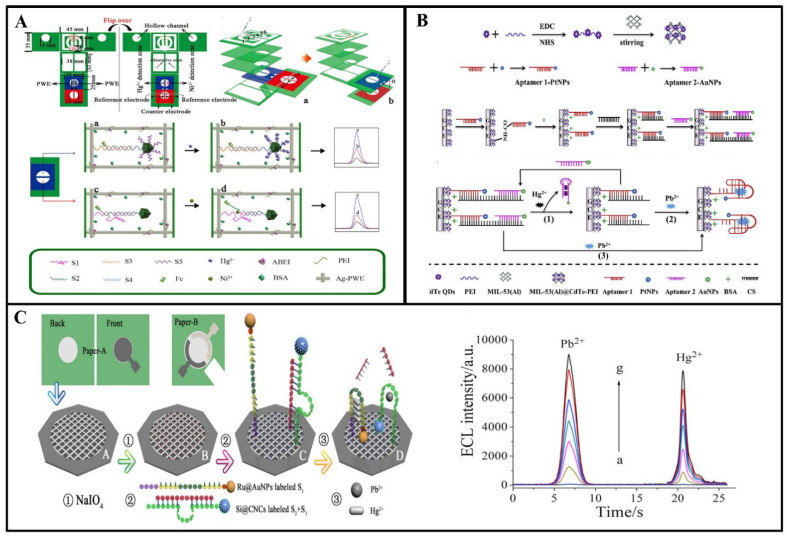
(**A**) Three-dimensional foldable fabrication procedure and detection of self-cleaning paper-based ECL biosensors [110]; (**B**) fabrication of MIL-53(Al)@CdTe-PEI fast sensor and detection of Hg^2+^ and Pb^2+^ [111]; (**C**) illustration depicting the 3D paper-based ECL device designed for the detection of Pb^2+^ and Hg^2+^. The back side of the paper is the waxed patterned paper work area: ① spotting of the paper with NaIO_4_; ② immobilization of Hg^2+^ with DNA strands labeled with Ru@AuNPs and Pb^2+^ with DNA strands labeled with Si@CNCs; and ③ trapping with Pb^2+^ and Hg^2+^. Reprinted with permission from Ref [113].

In the environment, the presence of metal ions spans air, water, soil, food, and even biological systems. Therefore, there is a demand for research and development of sensors capable of detecting two or more metal ions simultaneously. Separating the working area based on a microfluidic device enables simultaneous detection of multiple metal ions. For example, Zhang et al. fabricated a three-dimensional (3D) microfluidic-based analytical device for simultaneous ECL detection of Pb^2+^ and Hg^2+^ [110]. The silica nanoparticles of Ru(bpy)_3_^2+^ AuNP aggregates (Ru@AuNPs) and carbon nanocrystals (CNCs) (Si@CNCs) were used as terminal ECL tags and labeled with aptamers, respectively (Figure 11A). By leveraging the distinct reaction potentials of Si@CNCs and Ru@AuNPs, it becomes feasible to simultaneously detect Pb^2+^ and Hg^2+^ with impressive detection limits of 10 pM and 0.2 nM, respectively. Ultimately, this straightforward and cost-effective detection technique has been effectively utilized for the detection of Pb^2+^ and Hg^2+^ in lake water and human serum samples. This device provides the potential for high-throughput and rapid detection of metal ions. This is illustrated in Figure 11B. Yu et al. have innovatively integrated multiple fluidic channels and hollow structures on a paper substrate to realize the construction of an ECL biosensor, which is equipped with two working electrodes for signal acquisition, as well as effective cleaning to achieve highly sensitive detection of Ni^2+^ and Hg^2+^ [111]. This study dramatically reduced the testing time and provided new perspectives for the design of future paper-based metal ion diagnostic devices.

At the same time, functionalized nanomaterials and specific aptamers can also be used for highly selective detection of multiple metal ions. The team led by Hang created metal nanoclusters (NCs) using various biological macromolecules (such as proteins and DNA), thiol ligands, and polymers, resulting in NCs with diverse photoluminescence (PL) characteristics. They conducted research on the utilization of these NCs for the selective detection of heavy metal ions in water samples [112]. Feng et al. prepared a novel ECL sensor for multiple determination of Hg^2+^ and Pb^2+^ by using a metal nanoparticle-labeled T-rich aptamer and G-rich aptamer (Figure 11C) [113]. The detection mechanism is as follows: The prepared MIL-53 (Al)@CdTe QD composite is used as an ECL tag, and one end of the complementary chain chitosan hybridizes with the nucleic acid aptamer 1-PtNPs, and the other end hybridizes with the aptamer 2-AuNPs. Aptamer 1 binds with Pb^2+^ to create a nucleic acid aptamer that adopts a G-quadruplex structure. Aptamer 2 can establish a sandwich structure involving T-Hg, allowing the simultaneous detection of both ions by observing alterations in ECL intensity. The limit of detection (LOD) for Hg^2+^ is 4.1 pM, while that for Pb^2+^ is 24 pM.

## 5. Conclusions and Perspectives

Electrochemiluminescence (ECL) biosensing technology holds great promise in the field of heavy metal ion detection. This review extensively explores the composition and detection principles of ECL luminescent biosensors, emphasizing the crucial role of engineered ECL technology in achieving highly sensitive detection of heavy metals. Additionally, engineered ECL biosensors based on nanomaterial signal amplification are introduced as important tools for quantifying trace amounts of heavy metal and metal-like ions in various environmental and biological samples. Leveraging the programmability of DNA molecules, this review combines hybridization chain reaction (HCR), rolling circle amplification (RCA), entropy-driven catalysis (EDC), and clustered regularly interspaced short palindromic repeat (CRISPR) technologies as signal amplification strategies for heavy metal ion detection. Both nanomaterial-based and bioelement-based signal amplification strategies enhance sensor performance and reliability, better meeting monitoring requirements. Furthermore, emerging signal amplification technologies, such as dual-mode detection, paper-based microfluidic devices, and the use of smart materials for simultaneous detection of multiple ions, present broad prospects for future research and applications [114].

In the future, research in the field of ECL (electrochemiluminescence) bio-sensing based on heavy metal ions may need to address several challenges and potential limitations to ensure accurate identification and measurement of target ions in practical applications. The design and construction of engineered ECL biosensors should focus on the following aspects:

**(1) Multi-ion detection**: Expanding the application of engineered ECL biosensors to multi-ion detection, which is not limited to one or two heavy metal ions. Future research should be devoted to the design of multiplexed sensor arrays for the simultaneous monitoring of many different types of heavy metal ions. This will enhance the versatility of the sensor, especially for the analysis of environmental and biological samples, in order to more fully understand the presence and concentration of heavy metal ions.

**(2) Portability and miniaturization**: Although many ECL biosensors already have a certain degree of portability, the volume and weight of the supporting equipment may restrict their practical application. Future work can focus on designing more compact and miniaturized support devices to facilitate field testing and real-time monitoring. This will help to expand the applicability of engineered ECL biosensors and enable more people to use the technology easily.

**(3) Introducing new technologies**: For example, combining CRISPR/Cas gene editing technology with engineered ECL sensors for more accurate heavy metal ion detection and signal amplification. This combination will help to improve the specificity and accuracy of the sensor for better environmental and public health.

**(4) Cost effectiveness**: Reducing the preparation and application costs of engineered electrochemiluminescence biosensors to make them more economical and effective for wider promotion and use.

These improvements will contribute to the wider application of engineered ECL biosensors, address the challenges of heavy metal ion monitoring, and maintain the environment and public health. At the same time, electrochemical luminescence biosensor technology will be commercially applied in more social fields to ensure its reliable role in social health and environmental protection.

## References

[1] Rai P.K., Lee S.S., Zhang M., Tsang Y.F., Kim K.H. (2019). Heavy metals in food crops: Health risks, fate, mechanisms, and management. Environ. Int..

[2] Li L., Zhao W., Wang Y., Liu X., Jiang P., Luo L., Bi X., Meng X., Niu Q., Wu X. (2023). Gold nanocluster-confined covalent organic frameworks as bifunctional probes for electrochemiluminescence and colorimetric dual-response sensing of Pb^2+^. J. Hazard. Mater..

[3] Li Z., Liang Y., Hu H., Shaheen S.M., Zhong H., Tack F.M., Wu M., Li Y.-F., Gao Y., Rinklebe J. (2021). Speciation, transportation, and pathways of cadmium in soil-rice systems: A review on the environmental implications and remediation approaches for food safety. Environ. Int..

[4] Tepanosyan G., Sahakyan L., Belyaeva O., Asmaryan S., Saghatelyan A. (2018). Continuous impact of mining activities on soil heavy metals levels and human health. Sci. Total Environ..

[5] Zhou L., Li S., Li F. (2022). Damage and elimination of soil and water antibiotic and heavy metal pollution caused by livestock husbandry. Environ. Res..

[6] Nan X., Huyan Y., Li H., Sun S., Xu Y. (2021). Reaction-based fluorescent probes for Hg^2+^, Cu^2+^ and Fe^3+^/Fe^2+^. Coord. Chem. Rev..

[7] Mulenos M., Liu J., Lujan H., Guo B., Lichtfouse E., Sharma V., Sayes C. (2020). Copper, silver, and titania nanoparticles do not release ions under anoxic conditions and release only minute ion levels under oxic conditions in water: Evidence for the low toxicity of nanoparticles. Environ. Chem. Lett..

[8] LHe L., Zhong H., Liu G., Dai Z., Brookes P.C., Xu J. (2019). Remediation of heavy metal contaminated soils by biochar: Mechanisms, potential risks and applications in China. Environ. Pollut..

[9] Zhang M., Ye J., Li C., Xia Y., Wang Z., Feng J., Zhang X. (2019). Cytomembrane-Mediated Transport of Metal Ions with Biological Specificity. Adv. Sci..

[10] Medici S., Peana M., Pelucelli A., Zoroddu M.A. (2021). An updated overview on metal nanoparticles toxicity. Semin. Cancer Biol..

[11] Letchumanan D., Sok S.P.M., Ibrahim S., Nagoor N.H., Arshad N.M. (2021). Plant-Based Biosynthesis of Copper/Copper Oxide Nanoparticles: An Update on Their Applications in Biomedicine, Mechanisms, and Toxicity. Biomolecules.

[12] Soares E., Soares H. (2021). Harmful effects of metal(loid) oxide nanoparticles. Appl. Microbiol. Biotechnol..

[13] Boonta W., Talodthaisong C., Sattayaporn S., Chaicham C., Chaicham A., Sahasithiwat S., Kangkaew L., Kulchat S. (2020). The synthesis of nitrogen and sulfur co-doped graphene quantum dots for fluorescence detection of cobalt(ii) ions in water. Mater. Chem. Front..

[14] Wang W., Lu Y.-C., Huang H., Wang A.-J., Chen J.-R., Feng J.-J. (2014). Solvent-free synthesis of sulfur-and nitrogen-co-doped fluorescent carbon nanoparticles from glutathione for highly selective and sensitive detection of mercury(II) ions. Sens. Actuators B Chem..

[15] Wang Y., Kim S.-H., Feng L. (2015). Highly luminescent N, S- Co-doped carbon dots and their direct use as mercury(II) sensor. Anal. Chim. Acta.

[16] Awual R., Khraisheh M., Alharthi N.H., Luqman M., Islam A., Karim M.R., Rahman M.M., Khaleque A. (2018). Efficient detection and adsorption of cadmium(II) ions using innovative nano-composite materials. Chem. Eng. J..

[17] Chen S.-Y., Li Z., Li K., Yu X.-Q. (2021). Small molecular fluorescent probes for the detection of lead, cadmium and mercury ions. Coord. Chem. Rev..

[18] Domaille D.W., Que E.L., Chang C.J. (2008). Synthetic fluorescent sensors for studying the cell biology of metals. Nat. Chem. Biol..

[19] Zhao G., Liu G. (2019). Electrochemical Deposition of Gold Nanoparticles on Reduced Graphene Oxide by Fast Scan Cyclic Voltammetry for the Sensitive Determination of As(III). Nanomaterials.

[20] Dalal A., Bebarta A., Paul A., Barman R., Nunzi J.-M., Tameev A., Mahapatra R., Mondal A. (2023). Ion-Sensitive Three-Terminal Device as a Universal Hybrid Platform With TiO_2_ Nanowires on the Channel. IEEE Sens. J..

[21] Thakkar S., Dumée L.F., Gupta M., Singh B.R., Yang W. (2021). Nano-Enabled sensors for detection of arsenic in water. Water Res..

[22] Pereira L., Gonçalves I., da Silva J. (2004). Development of methodologies to determine aluminum, cadmium, chromium and lead in drinking water by ET AAS using permanent modifiers. Talanta.

[23] Byers H.L., McHenry L.J., Grundl T.J. (2019). XRF techniques to quantify heavy metals in vegetables at low detection limits. Food Chem. X.

[24] Zhang N., Suleiman J.S., He M., Hu B. (2008). Chromium(III)-imprinted silica gel for speciation analysis of chromium in environmental water samples with ICP-MS detection. Talanta.

[25] Lai S., Jin Y., Shi L., Zhou R., Li Y. (2023). Fluorescence Sensing Mechanisms of Versatile Graphene Quantum Dots toward Commonly Encountered Heavy Metal Ions. ACS Sens..

[26] Thirumalai M., Kumar S., Prabhakaran D., Sivaraman N., Maheswari M. (2018). Dynamically modified C_18_ silica monolithic column for the rapid determinations of lead, cadmium and mercury ions by reversed-phase high-performance liquid chromatography. J. Chromatogr. A.

[27] Li Y., Liu M.-L., Liang W.-B., Zhuo Y., He X.-J. (2023). Spherical nucleic acid enzyme programmed network to accelerate CRISPR assays for electrochemiluminescence biosensing applications. Biosens. Bioelectron..

[28] Kumar A., Jain D., Bahuguna J., Bhaiyya M., Dubey S.K., Javed A., Goel S. (2023). Machine learning assisted and smartphone integrated homogeneous electrochemiluminescence biosensor platform for sample to answer detection of various human metabolites. Biosens. Bioelectron..

[29] Liu Y., Cai Z., Sheng L., Ma M., Wang X. (2020). A magnetic relaxation switching and visual dual-mode sensor for selective detection of Hg^2+^ based on aptamers modified Au@Fe_3_O_4_ nanoparticles. J. Hazard. Mater..

[30] Qin X., Zhang X., Wang M., Dong Y., Liu J., Zhu Z., Li M., Yang D., Shao Y. (2018). Fabrication of Tris(bipyridine)ruthenium(II)-Functionalized Metal-Organic Framework Thin Films by Electrochemically Assisted Self-Assembly Technique for Electrochemiluminescent Immunoassay. Anal. Chem..

[31] Li J., Jia H., Ren X., Li Y., Liu L., Feng R., Ma H., Wei Q. (2022). Dumbbell Plate-Shaped AIEgen-Based Luminescent MOF with High Quantum Yield as Self-Enhanced ECL Tags: Mechanism Insights and Biosensing Application. Small.

[32] Palaniappan P., Krishnakumar N., Vadivelu M. (2009). Bioaccumulation of lead and the influence of chelating agents in *Catla catla* fingerlings. Environ. Chem. Lett..

[33] Kim H.K., Nguyen P.T., Kim M.I., Kim B.C. (2022). Aptamer-functionalized and silver-coated polydopamine-copper hybrid nanoflower adsorbent embedded with magnetic nanoparticles for efficient mercury removal. Chemosphere.

[34] Chen Y., Zhao P., Hu Z., Liang Y., Han H., Yang M., Luo X., Hou C., Huo D. (2023). Amino-functionalized multilayer Ti3C2Tx enabled electrochemical sensor for simultaneous determination of Cd^2+^and Pb^2+^ in food samples. Food Chem..

[35] Liu M., Hong Y., Duan X., Zhou Q., Chen J., Liu S., Su J., Han L., Zhang J., Niu B. (2023). Unveiling the metal mutation nexus: Exploring the genomic impacts of heavy metal exposure in lung adenocarcinoma and colorectal cancer. J. Hazard. Mater..

[36] Ren W., Huang P.-J., de Rochambeau D., Moon W., Zhang J., Lyu M., Wang S., Sleiman H., Liu J. (2020). Selection of a metal ligand modified DNAzyme for detecting Ni^2+^. Biosens. Bioelectron..

[37] Falcone E., Okafor M., Vitale N., Raibaut L., Sour A., Faller P. (2021). Extracellular Cu^2+^ pools and their detection: From current knowledge to next-generation probes. Coord. Chem. Rev..

[38] Chen Y., Zhao P., Liang Y., Liu Y., Zhao J., Hou J., Hou C., Huo D. (2023). A sensitive electrochemical sensor based on 3D porous melamine-doped rGO/MXene composite aerogel for the detection of heavy metal ions in the environment. Talanta.

[39] Yu D., Li R., Rong K., Fang Y., Liu L., Yu H., Dong S. (2023). A novel, environmentally friendly dual-signal water toxicity biosensor developed through the continuous release of Fe^3+^. Biosens. Bioelectron..

[40] Ali H., Khan E. (2018). Bioaccumulation of non-essential hazardous heavy metals and metalloids in freshwater fish. Risk to human health. Environ. Chem. Lett..

[41] Li X., Tan X., Yan J., Hu Q., Wu J., Zhang H., Chen X. (2016). A sensitive electrochemiluminescence folic acid sensor based on a 3D graphene/CdSeTe/Ru(bpy)_3_^2+^-doped silica nanocomposite modified electrode. Electrochim. Acta.

[42] Wang C., Zhang N., Wei D., Feng R., Fan D., Hu L., Wei Q., Ju H. (2019). Double electrochemiluminescence quenching effects of Fe_3_O_4_@PDA-Cu_x_O towards self-enhanced Ru(bpy)_3_^2+^ functionalized MOFs with hollow structure and it application to procalcitonin immunosensing. Biosens. Bioelectron..

[43] Bhardwaj J., Chaudhary N., Kim H., Jang J. (2019). Subtyping of influenza A H1N1 virus using a label-free electrochemical biosensor based on the DNA aptamer targeting the stem region of HA protein. Anal. Chim. Acta.

[44] Wang Y., Zhao G., Li X., Liu L., Cao W., Wei Q. (2018). Electrochemiluminescent competitive immunosensor based on polyethyleneimine capped SiO_2_ nanomaterials as labels to release Ru(bpy)_3_^2+^ fixed in 3D Cu/Ni oxalate for the detection of aflatoxin B1. Biosens. Bioelectron..

[45] Liu H., Chen C., Chen H., Mo L., Guo Z., Ye B., Liu Z. (2022). 2D-PROTACs with augmented protein degradation for super-resolution photothermal optical coherence tomography guided momentary multimodal therapy. Chem. Eng. J..

[46] Xiong C., Wang H., Liang W., Yuan Y., Yuan R., Chai Y. (2015). Luminescence-Functionalized Metal-Organic Frameworks Based on a Ruthenium(II) Complex: A Signal Amplification Strategy for Electrogenerated Chemiluminescence Immunosensors. Chem. A Eur. J..

[47] Zhao J., Jing P., Xue S., Xu W. (2017). Dendritic structure DNA for specific metal ion biosensor based on catalytic hairpin assembly and a sensitive synergistic amplification strategy. Biosens. Bioelectron..

[48] Liu H., Mo L., Chen H., Chen C., Wu J., Tang Z., Guo Z., Hu C., Liu Z. (2022). Carbon Dots with Intrinsic Bioactivities for Photothermal Optical Coherence Tomography, Tumor-Specific Therapy and Postoperative Wound Management. Adv. Healthc. Mater..

[49] Zhang B., Wei C. (2018). Highly sensitive and selective detection of Pb^2+^ using a turn-on fluorescent aptamer DNA silver nanoclusters sensor. Talanta.

[50] Tang W., Yu J., Wang Z., Jeerapan I., Yin L., Zhang F., He P. (2019). Label-free potentiometric aptasensing platform for the detection of Pb^2+^ based on guanine quadruplex structure. Anal. Chim. Acta.

[51] Liu Y., Deng Y., Li T., Chen Z., Chen H., Li S., Liu H. (2018). Aptamer-Based Electrochemical Biosensor for Mercury Ions Detection Using AuNPs-Modified Glass Carbon Electrode. J. Biomed. Nanotechnol..

[52] Lin S., Wang W., Hu C., Yang G., Ko C.-N., Ren K., Leung C.-H., Ma D.-L. (2017). The application of a G-quadruplex based assay with an iridium(III) complex to arsenic ion detection and its utilization in a microfluidic chip. J. Mater. Chem. B.

[53] Chen W., Ma J., Wu Z., Wang Z., Zhang H., Fu W., Pan D., Shi J., Ji Q. (2023). Cas12n nucleases, early evolutionary intermediates of type V CRISPR, comprise a distinct family of miniature genome editors. Mol. Cell.

[54] Liu L., Liu J.-W., Wu H., Wang X.-N., Yu R.-Q., Jiang J.-H. (2018). Branched Hybridization Chain Reaction Circuit for Ultrasensitive Localizable Imaging of mRNA in Living Cells. Anal. Chem..

[55] Mo L., He W., Li Z., Liang D., Qin R., Mo M., Yang C., Lin W. (2023). Recent progress in the development of DNA-based biosensors integrated with hybridization chain reaction or catalytic hairpin assembly. Front. Chem..

[56] Wang R., Zhao Y., Jie G. (2023). A novel DNA-quantum dot nanostructure electrochemiluminescence aptamer sensor by chain reaction amplification for rapid detection of trace Cd^2+^. Analyst.

[57] Xie X., Chai Y., Yuan Y., Yuan R. (2018). Dual triggers induced disassembly of DNA polymer decorated silver nanoparticle for ultrasensitive electrochemical Pb^2+^ detection. Anal. Chim. Acta.

[58] Wang Y., Xiao J., Lin X., Waheed A., Ravikumar A., Zhang Z., Zou Y., Chen C. (2023). A Self-Assembled G-Quadruplex/Hemin DNAzyme-Driven DNA Walker Strategy for Sensitive and Rapid Detection of Lead Ions Based on Rolling Circle Amplification. Biosensors.

[59] Zhou X., Su Q., Xing D. (2012). An electrochemiluminescent assay for high sensitive detection of mercury (II) based on isothermal rolling circular amplification. Anal. Chim. Acta.

[60] Yu L., Zhu L., Yan M., Feng S., Huang J., Yang X. (2021). Electrochemiluminescence Biosensor Based on Entropy-Driven Amplification and a Tetrahedral DNA Nanostructure for miRNA-133a Detection. Anal. Chem..

[61] Kim D., Garner O.B., Ozcan A., Di Carlo D. (2016). Homogeneous Entropy-Driven Amplified Detection of Biomolecular Interactions. ACS Nano.

[62] Zhu L., Zhu L., Zhang X., Yang L., Liu G., Xiong X. (2023). Programmable electrochemical biosensing platform based on catalytic hairpin assembly and entropy-driven catalytic cascade amplification circuit. Anal. Chim. Acta.

[63] Li J., Wang Y., Wang B., Lou J., Ni P., Jin Y., Chen S., Duan G., Zhang R. (2022). Application of CRISPR/Cas Systems in the Nucleic Acid Detection of Infectious Diseases. Diagnostics.

[64] Yao C., Ou J., Tang J., Yang D. (2022). DNA Supramolecular Assembly on Micro/Nanointerfaces for Bioanalysis. Accounts Chem. Res..

[65] Xu Y., Lv Z., Yao C., Yang D. (2022). Construction of rolling circle amplification-based DNA nanostructures for biomedical applications. Biomater. Sci..

[66] Talwar C.S., Park K.-H., Ahn W.-C., Kim Y.-S., Kwon O.S., Yong D., Kang T., Woo E. (2021). Detection of Infectious Viruses Using CRISPR-Cas12-Based Assay. Biosensors.

[67] Broughton J.P., Deng X., Yu G., Fasching C.L., Singh J., Streithorst J., Granados A., Sotomayor-Gonzalez A., Zorn K., Gopez A. (2020). Rapid Detection of 2019 Novel Coronavirus SARS-CoV-2 Using a CRISPR-based DETECTR Lateral Flow Assay. MedRxiv Prepr. Serv. Health Sci..

[68] Wang B., Zhang T., Yin J., Yu Y., Xu W., Ding J., Patel D.J., Yang H. (2021). Structural basis for self-cleavage prevention by tag:anti-tag pairing complementarity in type VI Cas13 CRISPR systems. Mol. Cell.

[69] Li Z., Li Z., Cheng X., Wang S., Wang X., Ma S., Lu Z., Zhang H., Zhao W., Chen Z. (2023). Intrinsic targeting of host RNA by Cas13 constrains its utility. Nat. Biomed. Eng..

[70] Luo M.L., Mullis A.S., Leenay R.T., Beisel C.L. (2015). Repurposing endogenous type I CRISPR-Cas systems for programmable gene repression. Nucleic Acids Res..

[71] Hoberecht L., Perampalam P., Lun A., Fortin J.-P. (2022). A comprehensive Bioconductor ecosystem for the design of CRISPR guide RNAs across nucleases and technologies. Nat. Commun..

[72] Broughton J., Deng X., Yu G., Fasching C., Servellita V., Singh J., Miao X., Streithorst J., Granados A., Sotomayor-Gonzalez A. (2020). CRISPR-Cas12-based detection of SARS-CoV-2. Nat. Biotechnol..

[73] Liu P.-F., Zhao K.-R., Liu Z.-J., Wang L., Ye S.-Y., Liang G.-X. (2021). Cas12a-based electrochemiluminescence biosensor for target amplification-free DNA detection. Biosens. Bioelectron..

[74] Yue Y., Wang S., Jin Q., An N., Wu L., Huang H. (2023). A triple amplification strategy using GR-5 DNAzyme as a signal medium for ultrasensitive detection of trace Pb2+based on CRISPR/Cas12a empowered electrochemical biosensor. Anal. Chim. Acta.

[75] Nikolaou P., Valenti G., Paolucci F. (2021). Nano-structured materials for the electrochemiluminescence signal enhancement. Electrochim. Acta.

[76] Liang W.-B., Zhuo Y., Zheng Y.-N., Xiong C.-Y., Chai Y.-Q., Yuan R. (2017). Electrochemiluminescent Pb^2+^-Driven Circular Etching Sensor Coupled to a DNA Micronet-Carrier. Acs Appl. Mater. Interfaces.

[77] Kesarkar S., Rampazzo E., Zanut A., Palomba F., Marcaccio M., Valenti G., Prodi L., Paolucci F. (2018). Dye-doped nanomaterials: Strategic design and role in electrochemiluminescence. Curr. Opin. Electrochem..

[78] Yu S., Du Y., Niu X., Li G., Zhu D., Yu Q., Zou G., Ju H. (2022). Arginine-modified black phosphorus quantum dots with dual excited states for enhanced electrochemiluminescence in bioanalysis. Nat. Commun..

[79] Yang L., Li J. (2023). Recent Advances in Electrochemiluminescence Emitters for Biosensing and Imaging of Protein Biomarkers. Chemosensors.

[80] Wang R., Wu H., Chen R., Chi Y. (2019). Strong Electrochemiluminescence Emission from Oxidized Multiwalled Carbon Nanotubes. Small.

[81] Tsai Y.-J., Kuo C.-L. (2022). The effect of N-doping on the electronic structure property and the li and Na storage capacity of graphene nanomaterials: A first-principles study. Electrochim. Acta.

[82] Li Q.-L., Ding S.-N. (2016). Multicolor electrochemiluminescence of core-shell CdSe@ZnS quantum dots based on the size effect. Sci. China-Chem..

[83] Anusuya T., Kumar V., Kumar V. (2021). Hydrophilic graphene quantum dots as turn-off fluorescent nanoprobes for toxic heavy metal ions detection in aqueous media. Chemosphere.

[84] Li J., Lu L., Kang T., Cheng S. (2016). Intense charge transfer surface based on graphene and thymine-Hg (II)-thymine base pairs for detection of Hg^2+^. Biosens. Bioelectron..

[85] Li L., Zhao W., Zhang J., Luo L., Liu X., Li X., You T., Zhao C. (2022). Label-free Hg(II) electrochemiluminescence sensor based on silica nanoparticles doped with a self-enhanced Ru(bpy)_3_^2+^-carbon nitride quantum dot luminophore. J. Colloid Interface Sci..

[86] Weng Z., Zhang Y., Zhang M., Huang Z., Chen W., Peng H. (2022). Gold Nanocluster Probe-Based Electron-Transfer-Mediated Electrochemiluminescence Sensing Strategy for an Ultrasensitive Copper Ion Detection. Anal. Chem..

[87] QZhou Q., Lin Y., Lin Y., Wei Q., Chen G., Tang D. (2016). Highly sensitive electrochemical sensing platform for lead ion based on synergetic catalysis of DNAzyme and Au-Pd porous bimetallic nanostructures. Biosens. Bioelectron..

[88] Liu F., Du F., Yuan F., Quan S., Guan Y., Xu G. (2022). Electrochemiluminescence bioassays based on carbon nitride nanomaterials and 2D transition metal carbides. Curr. Opin. Electrochem..

[89] Meskher H., Achi F. (2022). Electrochemical Sensing Systems for the Analysis of Catechol and Hydroquinone in the Aquatic Environments: A Critical Review. Crit. Rev. Anal. Chem..

[90] Eivazzadeh-Keihan R., Noruzi E.B., Chidar E., Jafari M., Davoodi F., Kashtiaray A., Gorab M.G., Hashemi S.M., Javanshir S., Cohan R.A. (2022). Applications of carbon-based conductive nanomaterials in biosensors. Chem. Eng. J..

[91] Peng Y., Li Y., Li L., Zhu J.-J. (2018). A label-free aptasensor for ultrasensitive Pb^2+^ detection based on electrochemiluminescence resonance energy transfer between carbon nitride nanofibers and Ru(phen)_3_^2+^. J. Hazard. Mater..

[92] Mo L., Liu H., Liu Z., Xu X., Lei B., Zhuang J., Liu Y., Hu C. (2022). Cascade Resonance Energy Transfer for the Construction of Nanoparticles with Multicolor Long Afterglow in Aqueous Solutions for Information Encryption and Bioimaging. Adv. Opt. Mater..

[93] Zhang M., Guo W. (2023). Simultaneous electrochemical detection of multiple heavy metal ions in milk based on silica-modified magnetic nanoparticles. Food Chem..

[94] Wang B., Zhao L., Li Y., Liu X., Fan D., Wu D., Wei Q. (2023). Porphyrin-based metal-organic frameworks enhanced electrochemiluminescence (ECL) by overcoming aggregation-caused quenching: A new ECL emitter for the detection of trenbolone. Anal. Chim. Acta.

[95] Liu S.-Q., Chen J.-S., Liu X.-P., Mao C.-J., Jin B.-K. (2023). An electrochemiluminescence aptasensor based on highly luminescent silver-based MOF and biotin-streptavidin system for mercury ion detection. Anal..

[96] Chen S., Ma H., Padelford J., Qinchen W., Yu W., Wang S., Zhu M., Wang G. (2019). Near Infrared Electrochemiluminescence of Rod-Shape 25-Atom AuAg Nanoclusters That Is Hundreds-Fold Stronger Than That of Ru(bpy)_3_ Standard. J. Am. Chem. Soc..

[97] D’Alton L., Nguyen P., Carrara S., Hogan C.F. (2021). Intense near-infrared electrochemiluminescence facilitated by energy transfer in bimetallic Ir-Ru metallopolymers. Electrochim. Acta.

[98] Zhou Y., He J., Zhang C., Li J., Fu X., Mao W., Li W., Yu C. (2020). Novel Ce(III)-Metal Organic Framework with a Luminescent Property To Fabricate an Electrochemiluminescence Immunosensor. ACS Appl. Mater. Interfaces.

[99] Han Q., Wang C., Liu P., Zhang G., Song L., Fu Y. (2021). Three kinds of porphyrin dots as near-infrared electrochemiluminescence luminophores: Facile synthesis and biosensing. Chem. Eng. J..

[100] Song X., Ren X., Zhao W., Zhao L., Wang S., Luo C., Li Y., Wei Q. (2022). A Portable Microfluidic-Based Electrochemiluminescence Sensor for Trace Detection of Trenbolone in Natural Water. Anal. Chem..

[101] Lin Y., Gritsenko D., Feng S., Teh Y.C., Lu X., Xu J. (2016). Detection of heavy metal by paper-based microfluidics. Biosens. Bioelectron..

[102] Li Y., Gao X., Fang Y., Cui B., Shen Y. (2023). Nanomaterials-driven innovative electrochemiluminescence aptasensors in reporting food pollutants. Co-ord. Chem. Rev..

[103] Hao T., Zhang C., Lin H., Wei W., Yang F., Wu Y., Niu L., Kang W., Guo Z. (2020). A One-Step Dual-Mode Aptasensor for Subnanomolar Detection of Lead Ions Based on Electrochemiluminescence and Fast Scan Voltammetry. J. Electrochem. Soc..

[104] Fu Y., Du C., Zhang Q., Xiao K., Zhang X., Chen J. (2022). Colorimetric and Photocurrent-Polarity-Switching Photoelectrochemical Dual-Mode Sensing Platform for Highly Selective Detection of Mercury Ions Based on the Split G-Quadruplex-Hemin Complex. Anal. Chem..

[105] Ma Y., Yu Y., Mu X., Yu C., Zhou Y., Chen J., Zheng S., He J. (2021). Enzyme-induced multicolor colorimetric and electrochemiluminescence sensor with a smartphone for visual and selective detection of Hg^2+^. J. Hazard. Mater..

[106] Maharjan S., Cecen B., Zhang Y. (2020). 3D Immunocompetent Organ-on-a-Chip Models. Small Methods.

[107] Wang Z., Pan J., Li Q., Zhou Y., Yang S., Xu J., Hua D. (2020). Improved AIE-Active Probe with High Sensitivity for Accurate Uranyl Ion Monitoring in the Wild Using Portable Electrochemiluminescence System for Environmental Applications. Adv. Funct. Mater..

[108] Ma S., Zhao W., Zhang Q., Zhang K., Liang C., Wang D., Liu X., Zhan X. (2023). A portable microfluidic electrochemical sensing platform for rapid detection of hazardous metal Pb^2+^ based on thermocapillary convection using 3D Ag-rGO-f-Ni(OH)_2_/NF as a signal amplifying element. J. Hazard. Mater..

[109] Zhu L., Lv X., Li Z., Shi H., Zhang Y., Zhang L., Yu J. (2021). All-sealed paper-based electrochemiluminescence platform for on-site determination of lead ions. Biosens. Bioelectron..

[110] Zhang M., Ge L., Ge S., Yan M., Yu J., Huang J., Liu S. (2013). Three-dimensional paper-based electrochemiluminescence device for simultaneous detection of Pb^2+^ and Hg^2+^ based on potential-control technique. Biosens. Bioelectron..

[111] Huang Y., Li L., Zhang Y., Zhang L., Ge S., Yu J. (2019). Auto-cleaning paper-based electrochemiluminescence biosensor coupled with binary catalysis of cubic Cu_2_O-Au and polyethyleneimine for quantification of Ni^2+^ and Hg^2+^. Biosens. Bioelectron..

[112] Nain A., Tseng Y.-T., Lin Y.-S., Wei S.-C., Mandal R.P., Unnikrishnan B., Huang C.-C., Tseng F.-G., Chang H.-T. (2020). Tuning the photoluminescence of metal nanoclusters for selective detection of multiple heavy metal ions. Sens. Actuators B Chem..

[113] Feng D., Li P., Tan X., Yeyu W., Wei F., Du F., Ai C., Luo Y., Chen Q., Han H. (2020). Electrochemiluminescence aptasensor for multiple determination of Hg^2+^ and Pb^2+^ ions by using the MIL-53(Al)@CdTe-PEI modified electrode. Anal. Chim. Acta.

[114] Liu H., Liu Z., Wang Y., Xiao J., Liu X., Jiang H., Wang X. (2023). Intracellular Liquid-Liquid Phase Separation Induces Tunable Anisotropic Nanocrystal Growth for Multidimensional Analysis. Adv. Funct. Mater..

